# Health-related quality of life and health preference of Chinese patients with diabetes mellitus managed in primary care and secondary care setting: decrements associated with individual complication and number of complications

**DOI:** 10.1186/s12955-017-0699-4

**Published:** 2017-06-13

**Authors:** Fangfang Jiao, Carlos King Ho Wong, Rita Gangwani, Kathryn Choon Beng Tan, Sydney Chi Wai Tang, Cindy Lo Kuen Lam

**Affiliations:** 1 Department of Family Medicine and Primary Care, Li Ka Shing Faculty of Medicine, The University of Hong Kong, 3/F, Ap Lei Chau Clinic, 161 Ap Lei Chau Main Street, Ap Lei Chau, Hong Kong, Hong Kong; 2 Department of Ophthalmology, Li Ka Shing Faculty of Medicine, The University of Hong Kong, Level 3 Block B Cyberport 4, 100 Cyberport Road, Hong Kong, Hong Kong; 3 Department of Medicine, Li Ka Shing Faculty of Medicine, The University of Hong Kong, 21 Sassoon Road, Hong Kong, Hong Kong

**Keywords:** Diabetes mellitus, Health-related quality of life, Health preference, Heart disease, Stroke, Microvascular complications

## Abstract

**Background:**

Health-related quality of life (HRQoL) and health preference of patients with diabetes mellitus (DM) are essential in health economic evaluations but data on Chinese population is rare. This study aims to evaluate HRQoL and health preference of diabetic patients with different diabetic complications in Chinese population.

**Methods:**

A cross-sectional study was conducted in 1275 patients with DM, including 518 subjects with various DM-related complications. HRQoL and health preference were estimated using SF-12 and SF-6D questionnaires, respectively. Disease status of DM and complications were identified from documented clinical diagnosis. Multivariable regression was used to investigate the effects of specific complications on HRQoL and health preference, adjusting for socio-demographic and clinical parameters.

**Results:**

The presence of any diabetic complication was associated with lower physical component summary (−3.81 points, *P* < 0.01), and end-stage renal disease (ESRD) showed greatest reduction (−7.05 points, *P* < 0.01). Mental component summary and mental health (MH) scores were not decreased in any of the diabetic complications. The health preference score for diabetic subjects without complications was 0.882 (95% CI, 0.778 to 0.989). The reductions of health preference score were significant for stroke (−0.042, 95% CI -0.072 to −0.012), ESRD (−0.055, 95% CI -0.093 to −0.017), and sight-threatening diabetic retinopathy (STDR) (−0.043, 95% CI -0.075 to −0.010), while heart disease had an insignificant reduction (−0.017, 95% CI -0.042 to 0.008).

**Conclusions:**

The presence of any of the four major diabetic complications (heart disease, stroke, ESRD and STDR) was associated with lower HRQoL and health preference scores. Findings of this study facilitated the cost-effectiveness studies of alternative management strategies for prevention of diabetic complications in Chinese population.

**Electronic supplementary material:**

The online version of this article (doi:10.1186/s12955-017-0699-4) contains supplementary material, which is available to authorized users.

## Background

Diabetes mellitus (DM) is highly prevalent and incident chronic disease worldwide [1]. The number of diabetic patients in China had reached 98 million in 2013, accounting for a quarter of the total diabetic patients in the world [2]. With the substantial prolonging life expectancy in DM [3], health-related quality of life (HRQoL) is increasingly recognized as an important outcome of chronic disease, reflecting the subjective impact of a disease condition and related interventions on patient-reported outcomes. The effectiveness of an intervention on medical outcomes might be of interest to health care providers; however, from the patients’ viewpoint, outcomes are meaningful only if they can feel the positive changes in physical, emotional and social wellbeing, that is, health-related quality of life [4]. The self-perception of changes in HRQoL can also motivate patients to engage in DM self-management [4].

HRQoL is a useful tool in health outcomes research and health technology assessment. In the economic evaluation of DM management, quality-adjusted life years (QALYs) is a commonly used outcome measure to evaluate the effectiveness of the intervention in managing DM and its complications. QALYs are estimated by multiplying the number of years subjects spend in a particular health state by a preference weight associated with that health state, which was known as health preference. Health preference could be estimated by both direct and indirect methods. The direct methods were trials that ask subjects to score their preference for the specific health states, and included standard Gamble and time trade-off. Health preference could also be estimated by indirect methods, which ask subjects to define the health states they experience by their response to surveys about various aspects of their health. The responses are converted into a single score through specific scoring algorithms [5]. In this study, we used indirect methods to estimate the health preference by SF-6D through regression-based mapping [6].

Studies showed that DM highly impaired HRQoL [7–10]. However, few studies have differentiated the impacts due to DM alone and those from DM-related complications. It is reported that different diabetic complications may jeopardize different domains of HRQoL. Macrovascular complications (heart disease and stroke) were found to affect only physical domains of HRQoL in some studies [11–13], but often observed an impact on both physical and mental domains [14, 15]. In terms of microvascular complications (nephropathy, retinopathy and neuropathy), only the severe level of disease consistently showed lower scores in physical aspect of HRQOL [11–13]. However, most of these studies rely on self-reported diseases, therefore only complications with severe clinical manifestations were frequently studied. Few studies have examined the whole spectrum of complications, especially in asymptomatic patients using standardized clinical diagnosis. Cong et al. estimated the HRQoL among Chinese patients with T2DM in Tianjin by the diabetes-specific HRQoL questionnaire found that diabetic neuropathy, peripheral vascular disease (PVD) and coronary heart disease (CHD) were associated with decreased HRQoL. However, this study was conducted in a limited number of subjects (*N* = 174) that they did not study the impact of the whole spectrum of complications on HRQoL. Also, the disease-specific HRQoL questionnaire only provides one single score to measure the HRQoL that it could not tell the impact of complications on different HRQoL domains [16].

Many overseas studies have reported the impact of different DM-related complications on health preference [17–19]. Renal failure, heart failure and amputation were reported as the complications associated with most reduction in health preference [20]. Health preference seems sensitive to patients’ cultural and socioeconomic characteristics. Besides the discrepancies in age, sex, disease severity and case definitions, culture and population setting can greatly influence HRQOL utility perceptions [17]. Despite lying on the same metric of health preference measure, variations in health preference values were found in association with DM and its complications in different studies [21, 22], prompting that health preference is population specific. A study showed that the EQ-5D health preference for the same disease states were valuated differently between the UK subjects and the US subjects [23]. Among the 42 health states measured by SF-6D, the US mean valuations were higher than the UK for 39 health states with a mean difference of 0.11 (range: -0.01 to 0.25) [23]. Therefore, to accurately evaluate the effectiveness and cost-effectiveness of DM interventions in our Chinese population, local empirical data on HRQoL and health preference associated with DM and its complications are required. Luk et al. estimated the health preference of Chinese T2DM in Hong Kong by EQ- 5D using the UK tariff. Three diabetic complications were found to be associated with significant decrease in EQ-5D index, including nephropathy, neuropathy and cardiovascular disease, with decrements of 0.014, 0.063 and 0.034, respectively. This study did not differentiate the severity of microvascular complications and was based on the UK tariff [24]. A recent study reported the associations between self-reported diabetic complications and health preference measured by EQ-5D-5L. Heart disease and cerebrovascular disease were found to have decrements of 0.074 and 0.16. This study did not cover the whole spectrum of diabetic complications, such as nephropathy and the complications were self-reported [25]. Previous economic evaluation studies on DM management in Chinese population were limited by the lack of health preference data in Chinese diabetic patients [26–28].

To date, little is known about, to what extent, whether DM and various DM-related complications affect HRQoL and health preference in a Chinese population. The prevalence of DM has accelerated in Asia, and China is now among the countries with the highest diabetes prevalence in Asia with a prevalence of 11.6% among adult population [29]. Several unique factors in Asians, especially in Chinese, might contribute to the rapid increase in DM epidemic, including the “normal-weight metabolically obese” phenotype; high intake of refined carbohydrates (e.g., white rice); dramatically decreased physical activity levels; and poor nutrition in utero and in early life combined with overnutrition in later life [30]. This study aimed to evaluate HRQoL and health preference of DM patients without complications and those with major DM-related complications including heart diseases, stroke, chronic kidney disease and retinopathy, thus to provide the contemporary empirical data for economic evaluation of DM management in Chinese population.

## Methods

A cross-sectional study was conducted to measure the HRQoL and health preference for uncomplicated and complicated DM.

### Subjects

The study subjects included two convenience samples, one from primary care setting and another one from secondary care. The former composed of diabetic patients recruited from Hospital Authority primary care general outpatient clinics (GOPC) in all seven clusters in Hong Kong. From September 2012 to January 2013, patients with DM were recruited for evaluation of Patient-reported outcome as part of evaluation of two quality improvement programs namely, multidisciplinary Risk Assessment and Management Program for patients with Diabetes Mellitus (RAMP-DM) [31, 32] and Patient Empowerment Programme [33, 34]. We adopted the baseline data of these subjects to avoid the effects of the interventions. The inclusion criteria were 1) aged 18 or above; and 2) with documented diagnosis of DM (ICPC-2 code T89/T90: insulin dependent DM/ non-insulin dependent DM) in the clinical management system.

To complement the GOPC sample in whom most (77%) of them were free from complications, the latter one was sampled from diabetic patients with known complications attending secondary care hospital-based setting endocrinology and nephrology specialist clinics in the Queen Mary Hospital and ophthalmology specialist clinic in Grantham Hospital. The inclusion criteria were 1) aged 18 or above; 2) with documented diagnosis of DM (ICD-9- CM code 250: diabetes mellitus) in HA clinical management system (CMS); 3) Documented diagnosis of at least one of the following DM-related complications: a) heart disease (coronary heart disease and/ or heart failure), b) stroke, c) nephropathy, or d) diabetic retinopathy with or without blindness. Exclusion criteria for both samples were 1) inability to complete an interview due to cognitive impairment base on the judgement of the attending clinician; 2) too ill to carry out an interview; 3) patients with other serious conditions, including cancer, chronic lung disease and dementia.

Written consents were obtained from all recruited subjects. Each recruited subject was contacted through telephone within 1 month after recruitment dates by trained interviewers of the Social Science Research Center of The University of Hong Kong to complete the Chinese (HK) SF-12v2 Health Survey and a questionnaire on socio-demographics and private health services utilization.

**Fig. 1:**
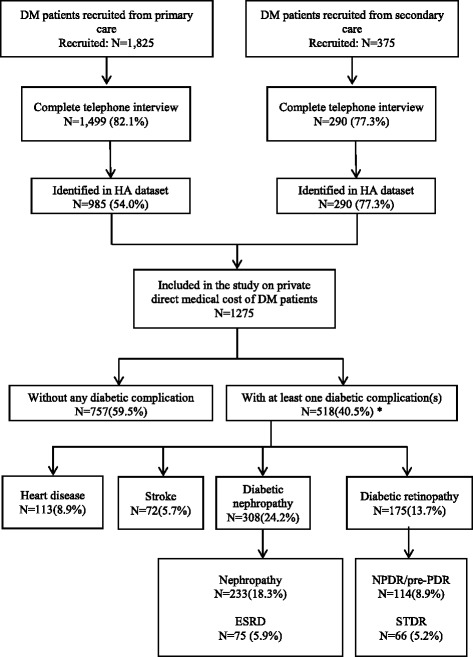
Flowchart of patient recruitment and interview. *Some subjects had more than one complications. CKD, chronic kidney disease; ESRD, end-stage renal disease; NPDR, non-proliferative diabetic retinopathy; PDR, proliferative diabetic retinopathy; STDR, sight-threatening diabetic retinopathy

As shown in Fig. 1, 1825 and 375 diabetic patients were recruited from primary care and hospital-based secondary care setting, respectively. Out of these, 1499 (82.1%) and 290 (77.3%) subjects completed the telephone interview, respectively. Among the subjects recruited from primary care, 514 subjects were excluded because of failure to be linked to the HA CMS to ascertain whether they had complications or not. The personal information of these 514 subjects were not well collected at the recruiting period. Therefore, we could not identify their clinical information in the Hospital Authority Clinical Management System. A total of 1275 subjects were included in the data analysis. Among these study subjects, 518 (40.6%) subjects were clinically diagnosed with at least one DM-related complication.

Ethics approval of the study has been sought by HKU/HA Institutional Review Board.

### Characteristics of study subjects

Socio-demographic characteristics including marital status and individual monthly income was self-reported by subjects during telephone interview. Clinical characteristics including age, sex, smoking status, duration of DM, HbA1c, blood pressure, lipid profiles and clinical diagnoses of diabetic complications were extracted from the Clinical Management System of the Hospital Authority in Hong Kong. The ICPC-2 and ICD-9-CM codes of diabetic complications are listed in Additional file 1: Table S1.

### Evaluation of Health-related Quality of Life and Health preference

The Chinese (Hong Kong) Short Form-12v2 (SF-12v2) was used to measure HRQoL. The SF-12v2 is a widely used generic HRQoL. The Chinese version has been validated and normed in the general Chinese population in Hong Kong [35], and measured HRQOL in local patients with DM [36, 37]. Eight domains of HRQoL are measured on a scale range from 0 to 100, including physical functioning (PF), role physical (RP), bodily pain (BP), general health (GH), vitality (VT), social functioning (SF), role emotional (RE) and mental health (MH). A higher score indicates better HRQoL. The eight domain scores are aggregated based on standardized weights and norm-based on the mean and SD of the Hong Kong population weights to calculate two summary scores, the physical (PCS) and mental component summary (MCS) scores [38].

Health preference was measured by the SF-6D that is derived from seven of twelve items of the SF-12v2 [39] calculated by the Hong Kong Chinese population-specific scoring algorithm [40, 41]. The theoretical range of SF-6D preference- based utility score ranges from 1 for full health to 0.315 for the worst possible health state for the Chinese Hong Kong population [40, 41].

### Statistical analysis

The SF-12v2 and SF-6D preference-based utility scores were calculated for each study subjects by the Hong Kong population specific algorithm [41]. Independent t-test and Tukey’s post hoc test were employed as appropriate to detect any difference in SF-12v2 and SF-6D scores for between groups and multiple groups comparison, respectively. Multivariable linear regressions were performed to estimate the independent effects associated with each subtype of DM- related complications on SF-12v2 and SF-6D scores, adjusting for the socio-demographic factors and clinical parameters. The regression was modeled using ordinary least squares (OLS) regression with robust standard errors. Although different methods were employed to regress the data due to the skewed distribution of HRQoL data [19, 42, 43], OLS regression is the commonest used method. Moreover, a study comparing OLS with other methods finds that OLS is a valid approach [44].

To determine whether there were differences in the HRQoL and health preference between diabetic patients and healthy population, we selected an age-sex stratified sample from our study subjects and an age-sex matched control sample randomly selected from a representative sample of 2533 Hong Kong general Chinese population, who had completed the SF-12v2 health survey in 2010 in a primary care service utilization study [38]. Among the 2533 general population, 888 subjects had self-reported chronic diseases, and they were excluded from matching. We did not perform a one-to-one matching because our study subjects were much older than the general population sample dataset, making a match pair for all the study subjects impossible. We were able to obtain a matched sub-sample of 220 pairs of our study subjects and the general population samples.

To determine the impact of number of complications on HRQoL and health preference, we divided subjects into three groups by the number of complications, which were subjects with no complication, with only one complication and with two or more complications. Tukey’s Post-hoc multiple comparisons were employed to compare the three groups.

All data analyses were performed using STATA Version 13.0 (StataCorp LP. College Station, Texus, US), and *P*- value less than 0.05 was considered statistically significant.

## Results

### Basic characteristics of study subjects

Table 1 shows the characteristics of the study subjects by disease status. The average age of study subjects without complications were generally younger than that of those with complications. The average duration of patients with uncomplicated DM was 7.76 years, which was shorter than those of subjects with any complication, whose duration of DM ranged from 10 to 17 years. Compared to subjects without any diabetic complications, subjects with complications all had lower TC and LDL-C, which might be due to intensive lipid-lowering treatments.

**Table 1:** Characteristics of study subjects

Variables	All study subjects	Without diabetic complication	Heart Diseases	Stroke	Diabetic nephropathy	ESRD	NPDR/pre-PDR	STDR
	(*N* = 1275)	(*N* = 757)	(*N* = 113)	(*N* = 72)	(*N* = 233)	(*N* = 75)	(*N* = 114)	(*N* = 66)
Age (years)	64.84 ± 10.26	63.38 ± 9.52	68.28 ± 8.54	70.22 ± 10.37	69.80 ± 10.09	65.17 ± 10.94	63.22 ± 10.52	63.96 ± 10.72
Male	46.75%	41.16%	63.72%	58.33%	46.78%	66.67%	60.53%	66.67%
Current smoker	19.85%	22.32%	16.35%	25.81%	13.84%	9.09%	10.19%	12.00%
Duration of DM (years)	9.35 ± 7.49	7.76 ± 6.27	14.26 ± 9.78	10.12 ± 8.25	11.08 ± 8.58	14.31 ± 16.89	17.08 ± 9.41	14.52 ± 9.61
SBP (mmHg)	138.30 ± 17.01	138.85 ± 16.83	133.73 ± 16.00	136.62 ± 16.27	137.79 ± 17.97	135.46 ± 16.89	136.02 ± 18.40	135.38 ± 19.15
DBP(mmHg)	76.38 ± 11.11	77.94 ± 10.18	71.87 ± 12.41	74.11 ± 10.85	73.92 ± 12.02	66.44 ± 11.13	73.00 ± 12.13	67.66 ± 12.01
HbA1c (%)	7.37 ± 1.25	7.38 ± 1.21	7.49 ± 1.32	7.10 ± 1.18	7.24 ± 1.06	7.06 ± 1.18	7.95 ± 1.30	6.92 ± 1.31
TC (mmol/L)	4.76 ± 1.36	4.91 ± 0.91	3.95 ± 0.91	4.51 ± 0.91	4.57 ± 1.05	4.32 ± 1.21	4.60 ± 3.27	4.31 ± 1.37
HDL-C (mmol/L)	1.30 ± 0.37	1.35 ± 0.36	1.09 ± 0.32	1.35 ± 0.42	1.24 ± 0.35	1.16 ± 0.37	1.17 ± 0.40	1.15 ± 0.40
LDL-C (mmol/L)	2.74 ± 0.86	2.87 ± 0.81	2.12 ± 0.78	2.56 ± 0.77	2.62 ± 0.92	2.40 ± 0.89	2.36 ± 0.94	2.34 ± 0.95
Triglyceride (mmol/L)	1.56 ± 1.03	1.53 ± 0.91	1.69 ± 1.21	1.36 ± 1.16	1.65 ± 0.98	1.88 ± 1.85	1.67 ± 1.19	1.91 ± 2.29
Marital status								
Single	6.27%	5.01%	6.19%	5.56%	6.87%	8.00%	12.28%	7.84%
Married	73.65%	75.99%	71.68%	65.28%	67.38%	78.67%	74.56%	82.35%
Divorce/Separated	5.18%	5.28%	4.42%	8.33%	5.58%	4.00%	4.39%	3.92%
Widow/Widower	14.90%	13.72%	17.70%	20.83%	20.17%	9.33%	8.77%	5.88%
Individual monthly income (HKD)								
< 2000	59.14%	60.16%	43.36%	62.50%	65.24%	68.00%	35.96%	72.55%
2000–14,999	31.45%	32.45%	40.71%	30.56%	25.32%	22.67%	42.11%	17.65%
15,000–29,999	6.75%	5.28%	14.16%	4.17%	6.44%	5.33%	13.16%	5.88%
> 29,999	2.67%	2.11%	1.77%	2.78%	3.00%	4.00%	8.77%	3.92%

Figures are expressed as Mean ± SD or % as appropriate

*DBP* diastolic blood pressure, *DM* diabetes mellitus, *HbA1c* glycated hemoglobin A1c, *HDL-C* high density lipid cholesterol, *LDL-C* low density lipid cholesterol, *SBP* systolic blood pressure, *TC* total cholesterol. *ESRD* end-stage renal disease, *NPDR* non-proliferative diabetic retinopathy, *PDR* proliferative diabetic retinopathy, *STDR* sight-threatening diabetic retinopathy

The age and sex between subjects included in the analysis and those excluded from the analysis were compared. Compared to subjects included in the analysis, the subjects excluded from the analysis were younger (63.87 ± 11.33 vs 64.84 ± 10.26, *P* = 0.037). The sex distribution was similar between these two groups (Additional file 1: Table S2).

### Comparison between Study Subjects and the General Healthy Population Sample

Table 2 presents the SF-12v2 and SF-6D health preference scores of diabetic patients and age-sex matched the general healthy population sample. The age-sex stratified subset of study subjects had similar SF-12v2 and SF-6D health preference scores as those of all study subjects. Compared to the general healthy population sample, study subjects had lower scores in SF-12v2 PF, BP, GH, VT, and PCS scores. The health preference score was similar between our study diabetic patients and the general healthy population sample (0.862 ± 0.113 versus 0.863 ± 0.102, *P* = 0.922).

**Table 2:** Comparison of health-related quality of life and SF-6D health preference between study subjects and age-sex matched general healthy population

Scales	All study subjects	Comparison		
		Sampled study subjects^a^	General healthy population	*P* value
	(*N* = 1275)	(*N* = 220)	(*N* = 220)	
Health related quality of life				
PF	80.46 ± 29.67	80.34 ± 28.72	86.82 ± 24.65	0.012
RP	80.65 ± 27.97	80.00 ± 26.40	84.32 ± 23.19	0.069
BP	79.00 ± 27.76	77.61 ± 28.40	82.73 ± 24.65	0.044
GH	36.56 ± 24.44	36.68 ± 24.26	51.77 ± 28.94	<0.001
VT	58.86 ± 29.96	56.93 ± 31.34	65.80 ± 26.67	0.002
SF	86.80 ± 26.26	82.61 ± 30.25	85.45 ± 23.62	0.273
RE	86.31 ± 22.97	83.13 ± 25.11	83.07 ± 20.94	0.979
MH	78.10 ± 21.20	76.76 ± 22.64	74.20 ± 20.22	0.212
PCS	45.89 ± 10.94	46.05 ± 10.41	50.33 ± 9.81	<0.001
MCS	54.97 ± 10.47	53.44 ± 11.78	52.75 ± 10.01	0.508
Health preference				
SF-6D score	0.868 ± 0.113	0.862 ± 0.113	0.863 ± 0.102	0.922

Figures are expressed as Mean ± SD

*BP* bodily pain, *GH* general health, *MCS* mental component summary score, *MH* mental health, *PCS* physical component summary score, *PF* physical functioning, *RE* role emotional, *RP* role physical, *SF* social functioning, *VT* vitality

^a^Proportional sampling from the total study subjects, all the items were not significantly different from total study subjects

### SF-12v2 Scores

The SF-12v2 scores of study subjects are shown in Table 3. Compared to the general healthy population sample, both uncomplicated and complicated diabetic patients had lower PCS. Uncomplicated diabetic patients had higher scores in SF, RE, MH and MCS, but lower scores in GH, VT and PCS. Complicated diabetic patients all showed lower scores in PF, BP, GH and VT than the general healthy population sample.

**Table 3:** Health-related quality of life of Chinese patients with diabetes mellitus with and without complication in comparison with general healthy population

HRQoL	General Healthy population	Without diabetic complication	Heart Diseases	Stroke	Diabetic Nephropathy	ESRD	NPDR /pre-PDR	STDR
	(*N* = 220)	(*N* = 757)	(*N* = 113)	(*N* = 72)	(*N* = 233)	(*N* = 75)	(*N* = 114)	(*N* = 66)
PF	86.82 ± 24.65	85.63 ± 25.83	70.54 ± 33.76*	73.26 ± 37.13*	70.92 ± 33.79*	64.53 ± 32.32*	**80.31 ± 27.64**	68.56 ± 33.77*
RP	84.32 ± 23.19	84.33 ± 25.56	73.78 ± 31.16*	69.79 ± 37.10*	76.66 ± 29.69*	70.00 ± 30.55*	78.95 ± 28.33	71.40 ± 31.15*
BP	82.73 ± 24.65	82.39 ± 25.18	71.46 ± 29.86*	68.40 ± 36.82*	77.36 ± 27.26*	69.00 ± 32.84*	76.75 ± 27.97*	67.80 ± 34.18*
GH	51.77 ± 28.94	**37.59 ± 23.91**	32.61 ± 24.74*	**33.68 ± 23.87**	**35.00 ± 24.89**	**35.07 ± 25.29**	**39.12 ± 26.63**	29.09 ± 23.32*
VT	65.80 ± 26.67	**61.68 ± 28.19**	49.56 ± 33.24*	50.00 ± 34.85*	**59.44 ± 29.21**	45.67 ± 30.31*	54.61 ± 33.02*	50.38 ± 32.07*
SF	85.45 ± 23.62	**89.81 ± 22.72**	77.88 ± 35.16*	78.82 ± 35.27*	86.27 ± 25.83	74.33 ± 34.38*	84.43 ± 28.88*	76.52 ± 35.05*
RE	83.07 ± 20.94	**88.42 ± 20.43**	80.20 ± 26.76*	79.17 ± 30.90*	86.86 ± 21.89	77.00 ± 29.64*	84.10 ± 26.52*	79.55 ± 28.81*
MH	74.20 ± 20.22	**78.78 ± 19.70**	75.88 ± 24.25	72.05 ± 26.66*	**78.54 ± 22.19**	**75.67 ± 22.78**	77.63 ± 23.19	74.43 ± 23.15
PCS	50.33 ± 9.81	**47.71 ± 9.75**	42.36 ± 12.20*	42.55 ± 14.27*	43.05 ± 12.08*	40.81 ± 11.52*	**45.75 ± 10.28**	41.12 ± 12.24*
MCS	52.75 ± 10.01	**55.30 ± 9.53**	52.96 ± 12.55*	52.05 ± 13.49*	**56.45 ± 10.73**	52.36 ± 12.43*	53.89 ± 11.64	52.94 ± 11.39

Figures in bold-*P* < 0.05 compare to general healthy population

Figures expressed as Mean ± SD

*BP* bodily pain, *ESRD* end-stage renal disease, *GH* general health, *MCS* mental component summary score, *MH* mental health, *PCS* physical component summary score, *PF* physical functioning, *RE*, role emotional, *RP* role physical, *SF* social functioning, *VT* vitality, *NPDR* non-proliferative diabetic retinopathy, *PDR* proliferative diabetic retinopathy, *STDR* sight-threatening diabetic retinopathy

*P < 0.05 compared to subjects without complications

Compared to uncomplicated diabetic patients, complicated diabetic patients except those with NPDR/pre-PDR, all had lower scores in PCS. Subjects with cardiovascular complications had lower scores in all the SF-12v2 domain scores except MH score for heart disease and GH for stroke. In terms of microvascular disease, subjects with NPDR or pre-PDR and moderate reduced renal function did not show much difference in SF-12v2 scores compared to subjects without complications, while subjects with STDR and ESRD showed lower means in most SF-12v2 scores.

After adjustment for socio-demographic, clinic parameters and co-existing complications, subjects with any diabetic complications had lower in all SF-12v2 domain scores except MH and MCS, compared to those without complications (Table 4). Stroke, ESRD, STDR were associated with lower scores in most SF-12v2 domains, while NPDR/pre-PDR was not associated with any significant decrease or increase in any SF-12v2 scores. Heart disease was associated with lower scores in GH and SF.

**Table 4:** Decrement in health-related quality of life associated with the presence of diabetic complication and individual complication

HRQoL	Any diabetic complication^a^	Individual complications^b^					
		Heart disease	Stroke	Diabetic nephropathy	ESRD	NPDR/pre-PDR	STDR
PF	-10.61 ± 1.83*	−4.04 ± 3.15	−5.81 ± 3.75	−7.76 ± 2.19	−18.78 ± 4.83*	−0.04 ± 3.41	−14.41 ± 4.08*
RP	-7.19 ± 1.81*	−0.84 ± 3.14	−9.08 ± 3.74*	−3.48 ± 2.18	−12.06 ± 4.78*	−1.51 ± 3.39	−8.46 ± 4.07*
BP	-7.25 ± 1.81*	−3.00 ± 3.13	−11.02 ± 3.73*	−1.54 ± 2.18	−14.16 ± 4.77*	1.14 ± 3.38	−11.18 ± 4.06*
GH	-3.63 ± 1.63*	−5.02 ± 2.81¶	−2.77 ± 3.35	−2.38 ± 1.95	−8.39 ± 4.28*	5.03 ± 3.03	−4.41 ± 3.64
VT	-6.82 ± 1.97*	−4.99 ± 3.40	−4.92 ± 4.06	−1.46 ± 2.37	−17.88 ± 5.19*	−2.49 ± 3.67	−7.42 ± 4.41
SF	-5.96 ± 1.73*	−5.95 ± 2.97*	−7.29 ± 3.55*	−1.11 ± 2.07	−13.58 ± 4.54*	2.66 ± 3.21	−8.74 ± 3.86*
RE	-4.81 ± 1.48*	−2.91 ± 2.55	−6.67 ± 3.05*	−0.64 ± 1.78	−9.67 ± 3.90*	0.40 ± 2.76	−3.49 ± 3.31
MH	-1.44 ± 1.40	−1.04 ± 2.42	−4.32 ± 2.88	−0.26 ± 1.68	−2.90 ± 3.69	2.52 ± 2.61	−5.01 ± 3.13
PCS	-3.81 ± 0.69*	−1.40 ± 1.19	−3.05 ± 1.42*	−2.32 ± 0.83*	−7.05 ± 1.83*	−0.07 ± 1.29	−4.93 ± 1.55*
MCS	-0.86 ± 0.68	−1.44 ± 1.18	−2.15 ± 1.41	0.63 ± 0.82	−13.58 ± 1.81*	0.65 ± 1.28	−1.13 ± 1.53

Figures expressed as Mean ± SE

*BP* bodily pain, *ESRD* end-stage renal disease, *GH* general health, *MCS* mental component summary score, *MH* mental health, *PCS* physical component summary score, *PF* physical functioning, *RE* role emotional, *RP* role physical, *SE* standard error, *SF* social functioning, *VT* vitality. *NPDR* non-proliferative diabetic retinopathy, *PDR* proliferative diabetic retinopathy, *STDR* sight-threatening diabetic retinopathy

^a^Adjusted for age, sex, smoking status, duration of DM, SBP, DBP, HbA1c, TC, HDL-C, LDL-C, triglyceride, marital status and individual monthly income

^b^Adjusted for age, sex, smoking status, duration of DM, SBP, DBP, HbA1c, TC, HDL-C, LDL-C, triglyceride, marital status and individual monthly income and other complications

*P < 0.05, ¶ *P* = 0.074

### SF-6D Health Preference Scores

Table 5 shows the SF-6D health preference scores. Compared to the general healthy population sample, the uncomplicated diabetic patients had higher health preference score (0.882 ± 0.102 versus 0.863 ± 0.102, *P* = 0.025). Subjects with heart diseases, stroke, ESRD and STDR had lower SF-6D health preference scores than the general healthy population sample and uncomplicated diabetic patients.

**Table 5:** Decrement in SF-6D health preference score associated with the presence of diabetic complication and individual complication

	SF-6D health preference scores	Compare to general population sample	Compare to subjects without complications	Decrements of specific complications^a^		
	Mean ± SD	*P* value	*P* value	Estimated coefficients	95% CI	*P* value
General healthy population (*N* = 220)	0.863 ± 0.102	NA	NA			
Without diabetic complication (*N* = 757)	0.882 ± 0.102	0.025	NA			
Individual complications						
Heart Diseases (*N* = 113)	0.834 ± 0.131	0.029	<0.001	−0.017	(−0.042,0.008)	0.190
Stroke (*N* = 72)	0.822 ± 0.150	0.01	<0.001	−0.042	(−0.072,-0.012)	0.005
Nephropathy (*N* = 233)	0.858 ± 0.117	0.029	<0.001	−0.011	(−0.029,0.006)	0.194
ESRD (*N* = 75)	0.820 ± 0.122	0.003	<0.001	−0.055	(−0.093,-0.017)	0.004
NPDR/pre-PDR (*N* = 114)	0.865 ± 0.124	0.881	0.139	0.004	(−0.024,0.032)	0.769
STDR (*N* = 66)	0.828 ± 0.130	0.023	<0.001	−0.043	(−0.075,-0.010)	0.010

Intercept: 0.883, 95% CI (0.778,0.989)

*ESRD* end-stage renal disease, *NPDR* non-proliferative diabetic retinopathy, *PDR* proliferative diabetic retinopathy, *STDR* sight-threatening diabetic retinopathy

^a^Adjusted for age, sex, smoking status, duration of DM, SBP, DBP, HbA1c, TC, HDL-C, LDL-C, triglyceride, marital status and individual monthly income and other complications

After adjustment for socio-demographic and clinical parameters, stroke, ESRD and STDR were associated with lower SF-6D health preference, with reductions of −0.042 (95% CI -0.072 to −0.012), −0.055 (95% CI -0.093 to −0.017) and −0.043 (95%CI -0.075 to −0.010), respectively. The marginal effect of heart disease became insignificant (−0.017, 95% CI -0.042 to 0.008).

### SF-12v2 and SF-6D Health Preference Scores by numbers of complications

Table 6 presents the associations between number of diabetic complications and SF-12v2 and SF-6D health preference scores. The impact on HRQoL increased with the development of complications especially if there were two or more. Subjects with only one complication showed lower scores in PF, RP, BP, SF, RE and PCS, as well as health preference than subjects without complications. Subjects with two or more complications had lower SF-12v2 PF, RP, BP, VT, PCS and SF-6D health preference scores than those with only one complication.

**Table 6:** Decrement in health-related quality of life and health preference associated with the number of diabetic complications

Scales	Number of complications				Effects of number of complications^b^		
	0 (*N* = 757)	1 (*N* = 379)	≥2 *N* = 139)	Multiple comparison^a^	1 (*N* = 379)	2 (*N* = 119)	≥3 (*N* = 18)
	(1) Mean ± SD	(2) Mean ± SD	(3) Mean ± SD	Compare (1), (2) &(3)	Mean ± SE	Mean ± SE	Mean ± SE
Health related quality of life							
PF	85.6 ± 25.8	75.6 ± 33.0	67.0 ± 32.7	(1) > (2); (1),(2) > (3)	−9.26 ± 1.97*	−12.51 ± 3.18*	−20.04 ± 7.51*
RP	84.3 ± 25.6	77.4 ± 29.4	71.0 ± 32.2	(1) > (2); (1),(2) > (3)	−5.78 ± 1.95*	−8.49 ± 3.16*	−8.51 ± 7.24
BP	82.4 ± 25.2	76.5 ± 29.8	69.1 ± 31.1	(1) > (2); (1),(2) > (3)	−5.43 ± 1.96*	−10.20 ± 3.16*	−6.26 ± 7.25
GH	37.6 ± 23.9	36.4 ± 25.4	31.9 ± 24.2	(1) > (3)	−2.75 ± 1.75	−6.00 ± 2.83*	−3.54 ± 6.49
VT	61.7 ± 28.2	57.3 ± 31.8	49.6 ± 31.7	(1) > (3); (1),(2) > (3)	−5.05 ± 2.12*	−7.53 ± 3.43*	−23.69 ± 7.88*
SF	89.8 ± 22.7	84.0 ± 28.9	79.0 ± 33.1	(1) > (2); (1) > (3)	−5.04 ± 1.86*	−6.85 ± 3.01*	−11.84 ± 6.90
RE	88.4 ± 20.4	84.5 ± 25.5	80.7 ± 26.8	(1) > (2); (1) > (3)	−4.52 ± 1.59*	−3.88 ± 2.57	−6.26 ± 5.90
MH	78.8 ± 19.7	78.4 ± 22.5	74.2 ± 24.4		−0.75 ± 1.51	−2.42 ± 2.43	−5.10 ± 5.58
PCS	47.7 ± 9.7	44.3 ± 11.7	41.1 ± 12.2	(1) > (2); (1),(2) > (3)	−3.11 ± 0.74*	−4.91 ± 1.20*	−5.55 ± 2.84*
MCS	55.3 ± 9.5	55.0 ± 11.3	53.4 ± 12.5		−0.63 ± 0.74	−0.66 ± 1.19	−4.07 ± 2.81
Health preference							
SF-6D score	0.882 ± 0.102	0.859 ± 0.120	0.827 ± 0.132	(1) > (2); (1),(2) > (3)	−0.022 ± 0.008*	−0.036 ± 0.013*	−0.065 ± 0.029*

*PF* physical functioning, *RP* role physical, *BP* bodily pain, *GH* general health, *VT* vitality, *SF* social functioning, *RE* role emotional, *MH* mental health, *PCS* Physical Component Summary score, *MCS* Mental Component Summary score

^a^ Significant difference between three groups by Tukey’s Post-hoc multiple comparisons

^b^ Adjusted for age, sex, smoking status, duration of DM, SBP, DBP, HbA1c, TC, HDL-C, LDL-C, triglyceride, marital status and individual monthly income and other complications

* *P* < 0.05

## Discussion

To the best of our knowledge, this was the first study to investigate the different effects of specific diabetic complications on HRQoL and health preference in a Chinese population. Patients with history of any of the four major DM-related complications (heart disease, stroke, ESRD and STDR) had lower SF-12v2 and SF-6D health preference scores than the general healthy population sample, as well as uncomplicated DM. After adjusted for socio-demographic and clinical parameters, heart disease was associated with lower GH and SF, but not SF-6D health preference. Stroke, ESRD and STDR were associated with lower PF, RP, BP, SF and PCS and SF-6D health preference. None of the complications were associated with lower MH and MCS.

Compared to the general healthy population sample, uncomplicated DM had lower scores in GH, VT and PCS, but higher scores in SF, RE, MH and MCS. As a result, the SF-6D health preference in uncomplicated DM was higher than that of the general healthy population sample. A few previous studies had compared the HRQoL of diabetic patients with non-diabetic patients. The findings on the impact of DM on HRQoL and health preference were inconclusive. A study in Singapore population showed that the HRQoL (measured by the SF-36 health survey) and SF-6D health preference score in diabetic patients without vascular complications were not significantly different from that of non-diabetic subjects [11]. A study in Spain also showed that health preference measured by the EQ-5D was not significantly lower in diabetic subjects without vascular complications compared to non-diabetic subjects [45]. Studies that did not adjust for diabetic complications found that diabetic patients had worse HRQoL than non-diabetic patients [7, 46–48]. There were several explanations for the discrepancy between our findings and previous studies. First, the lower HRQoL in previous studies might be due to the presence of DM-related complications in some study subjects. Second, all the previous studies used self-reported diagnosis of DM, while our study defined DM and complications by documented diagnosis in medical records by chart review. Third, subjects with DM tend to seek different ways of physical and emotional rehabilitation in person or in group. Their active response to their situation probably has a role in their better quality of life. Literature showed that both individual and group-based rehabilitation can improve DM patients HRQoL [49]. Forth, compared to our study subjects, subjects in these studies had lower proportion of being married [7, 46–48]. Our study showed that divorce/separated (coefficient − 0.041, *P* = 0.031, data not shown) and widowed (coefficient − 0.044, *P* = 0.009, data not shown) was negatively associated with health preference with married as reference group.

Although subjects with heart disease might not feel obvious body pain or role limitation, and there was minimal impairment of their physical functioning and role functioning (Tables 5 and 6), they still reported worse general health (decrement: -5.02, *P* = 0.062) and social functioning (decrement: -5.95, *P* < 0.05) probably because they were conscious and being cautious of life-threatening disease. Mild, reversible disease (nephropathy and NPDR/pre-PDR) did not lead to significant decrease in any SF-12v2 domains because they are asymptomatic. However, when the disease progressed to severer stage and became irreversible (ESRD and STDR), almost all the SF-12v2 domains had significant lower scores except MH. Therefore, it is important to screen and treat early these complications in DM subjects to preserve HRQoL.

None of the four major diabetic complications (heart disease, stroke, ESRD and STDR) were associated with lower mental aspect of HRQOL, which was consistent with overseas studies using SF-36 or SF-8 [11, 12, 50]. In our study, heart disease was found to have lower PCS than the general healthy population sample and subjects without complications, but the decrease was no longer significant after adjusting for clinical covariates and co-existing complications. Previous studies found that ischemic heart disease was associated with lower PCS in Singapore population [11, 13], the US [12] and UK [50]. Only one study in Singapore population (including 60% Chinese subjects) reported the impact of diabetic complications on 8 domains of HRQoL [11]. They found severe retinopathy was associated with lower scores in PF and RP, which was consistent to our study. Stroke was only associated with lower PF in Singapore population, while we found that patients with stroke had lower scores in RP, BP, SF, RE and PCS. There were only 10 subjects with stroke in the Singapore study, which could lead to wide variation in the scores.

Most previous studies on the health preference scores of diabetic patients employed EQ-5D [20, 51, 25, 52], one study in Singapore population (60% of the study subjects were Chinese) used SF-6D [11]. The SF-6D scores were 0.79 for non-diabetic subjects and 0.78 for uncomplicated DM in Singapore population [11], and the difference was not significant. The SF-6D score were lower than those found in our Hong Kong Chinese population, which were 0.86 for the general healthy population sample without DM and 0.88 for uncomplicated DM. Compared to uncomplicated DM, decrements of 0.02 to 0.03 in the SF-6D scores were observed for diabetic patients with coronary heart disease, stroke, severe retinopathy and severe nephropathy respectively in the Singapore study. Another study in Australian overweight or obese subjects used the SF-6D and found that DM was not associated lower SF-6D preference score but CHD decreased health preference by 0.054 [53]. In the Chinese population in our study, the decrements in health preference were 0.031, 0.051, 0.047 and 0.037 for subjects with heart disease, stroke, ESRD and STDR. The SF-6D scores in our study were calculated by the Hong Kong Chinese population-specific scoring algorithm [40, 41], while the Singapore study and Australia study adopted the algorithm derived from UK general population [54]. In regarding to sociodemographic parameters, the subjects in the Singapore study was younger than our subjects (48 ± 11 vs 65 ± 10 years-old), lower proportion of subjects were separated/divorced/widowed (5.7% vs 20.8%). Only 60% subjects were Chinese and 40% were Malay and India in the Singapore study. Our study showed that age was not significantly associated with changes in health preference while divorce/separated and widowed was negatively associated with health preference with married as reference group. The lower health preference in the Singapore study was not likely to be explained by the differences in sociodemographic characteristics. The differences in scoring algorithm and population composition might cause the differences in the health preference.

This study found that subjects with two or more complications showed significantly lower scores than those with only one complication in physical component scores and SF-6D score, but unexpectedly MH and MCS scores were not significantly decreased with the increase in the number of complications. A study in Norway population using EQ-5D also showed a marked difference in health preference scores for DM patients with only one complication (0.80) and subjects with two or more complications (0.64) [18].

There were several strengths in this study. First, the numbers of subjects with and without diabetic complications were large enough to detect the relative differences in HRQoL and health preference. Second, purposeful sampling enabled us to assess the impact of different complications on HRQoL and health preference. Third, disease status was defined by documented clinical diagnoses, which are more reliable than self-reported diseases. Fourth, comprehensive demographic and clinical parameters were included in the regressions to determine the independent associations between specific diabetic complications and health preference.

The limitations of our study should be considered when interpreting the study results. First, we did not differentiate T1DM and T2DM in this study because T2DM accounted for over 90% of all diabetic patients [2], although this is not likely to affect our conclusions. Second, it was a cross-sectional study in which only association but not causation could be established. Third, we used telephone survey to collect HRQoL which might select respondents with better HRQoL and biased towards higher HRQoL scores. The same bias should have been applied to all the subjects, so the relative differences among different disease states were less likely to be biased. Fourth, the drop-out rate in the GOPC sample was relatively high (46.0%) due to incomplete information collection during the recruit period for 514 subjects, although all of them completed the telephone interview. The high drop-out rate caused a reduction in the sample size. Since all of these 514 subjects were randomly lost due to accidental cause instead of refusal, this was not likely to cause selection bias. Fifth, education level is an important sociodemographic factor, but we did not include it into analysis due to high missing rate (25%). Living place area was an important sociodemographic factor to reflect the socioeconomic status, of which was not collected in telephone survey.

## Conclusions

The HRQoL and health preference of Chinese diabetic patients without complications were not worse than those of the general healthy population. The presence of any of the four major diabetic complications (heart disease, stroke, ESRD and STDR) was associated with significantly lower HRQoL and health preference scores. To preserve HRQOL in the long-term, preventing or delaying the onset of diabetic complications should be the target goal and quality indicator of DM management in primary care. The health preference scores of different DM-related disease states provided in this study would facilitate the cost-utility or cost-effectiveness studies of alternative management strategies for prevention of diabetic complications in Chinese population.

## Additional file

Additional file 1: Table S1. ICD-9CM, ICPC-2 Codes for Diabetes-related Complications. **Table S2.** Comparison of age and gender between the subjects included in analysis and those excluded from analysis. (DOCX 15 kb)

## Abbreviations

BP: Bodily pain
CHD: Coronary heart disease
DBP: Diastolic blood pressure
DM: Diabetes mellitus
ESRD: End-stage renal disease
GH: General health
HbA1c: Glycated hemoglobin A1c
HDL-C: High density lipid cholesterol
HRQoL: Health-related quality of life
LDL-C: Low density lipid cholesterol
MCS: Mental component summary score
MH: Mental health
NPDR: Non-proliferative diabetic retinopathy
PCS: Physical component summary score
PDR: Proliferative diabetic retinopathy
PF: Physical functioning
PVD: Peripheral vascular disease
QALY: Quality-adjusted life years
RE: Role emotional
RP: Role physical
SBP: Systolic blood pressure
SF: Social functioning
STDR: Sight-threatening diabetic retinopathy
TC: Total cholesterol
VT: Vitality
