# Supplementary material for: Health-related quality of life and health preference of Chinese patients with diabetes mellitus managed in primary care and secondary care setting: decrements associated with individual complication and number of complications
Source: Health Qual Life Outcomes. 2017 Jun 13;15:125. doi: 10.1186/s12955-017-0699-4 (PMC5470199; doi:10.1186/s12955-017-0699-4)
Supplement: Additional file 1: Table S1. — ICD-9CM, ICPC-2 Codes for Diabetes-related Complications. Table S2. Comparison of age and gender between the subjects included in analysis and those excluded from analysis. (DOCX 15 kb) [file 12955_2017_699_MOESM1_ESM.docx]

Table S1. ICD-9CM, ICPC-2 Codes for Diabetes-related Complications

| Disease | ICPC-2 Codes | | | ICD-9-CM Codes |
| --- | --- | --- | --- | --- |
| Heart disease | K74-K77 | | | 410.00-410.92, 411.0-411.89; 412; 413.0-413.9;414.0-414.9, 428.0-428.9, 798.1-798.9 |
| Stroke | K89-K91 | | | 430;431;432.0-432.9;433.00-433.91;434.00-434.91;435.0-435.9; 436; 437.0-437.9; 438.0-438.9. |
| NPDR/pre-PDR | F83 | | | 249.5, 250.5; 362.01; 362.03-362.06;365;366.41; |
| STDR | NA | | | 362.02;362.07 |
| Diabetic nephropathy | NA | | | 250.40-250.43; 249.40-249.41; 585.1-585.4;585.9;791.0 |
| ESRD | NA | | | 585.5-585.6; 586 |
|  | |  |  |  |

ESRD,end-stage renal disease; NPDR, non-proliferative diabetic retinopathy; PDR, proliferative diabetic retinopathy; STDR, sight threatening diabetic retinopathy.

Table S2. Comparison of age and gender between the subjects included in analysis and those excluded from analysis

| Variables | Include in analysis | Exclude from analysis | P value |
| --- | --- | --- | --- |
|  |  |  |  |
|  | (N=1275) | (N=925) |  |
| Age (years) | 64.84±10.26 | 63.87±11.33 | 0.037 |
| Male | 46.75% | 47.46% | 0.110 |
